# Physical training improves physical activity levels but is associated with amplification of sedentary behavior in older women

**DOI:** 10.3389/fpubh.2023.1180901

**Published:** 2023-06-02

**Authors:** Guilherme Lima de Oliveira, Andressa C. S. Sobrinho, Cicero Jonas R. Benjamim, Guilherme da Silva Rodrigues, Karine Pereira Rodrigues, Carlos Henrique Fernandes, Adriano Bruno Corrêa, Tamara Nascimento Felicio, Grace Angelica de Oliveira Gomes, Carlos Roberto Bueno Júnior

**Affiliations:** ^1^Escola de Educação Física e Esporte de Ribeirão Preto, Universidade de São Paulo (USP), Ribeirão Preto, SP, Brazil; ^2^Faculdade de Medicina de Ribeirão Preto, Universidade de São Paulo (USP), Ribeirão Preto, SP, Brazil; ^3^Departamento de Gerontologia da Universidade Federal de São Carlos (UFSCAR), São Carlos, SP, Brazil; ^4^Escola de Enfermagem de Ribeirão Preto da Universidade de São Paulo (USP), Ribeirão Preto, SP, Brazil

**Keywords:** sitting time, aging, physical aptitude, physical performance, sedentary behavior

## Abstract

Physical activity level (PAL) and sedentary behavior (SB) are independent predictors of mortality. It is unclear how these predictors interact with each other and health variables. Investigate the bidirectional relationship between PAL and SB, and their impact and health variables of women aged 60 to 70 years. One hundred forty-two older adults women (66.3 ± 2.9 years) considered insufficiently active were submitted to 14 weeks of multicomponent training (MT), multicomponent training with flexibility (TMF), or the control group (CG). PAL variables were analyzed by accelerometry and QBMI questionnaire, physical activity (PA) light, moderate, vigorous and CS by accelerometry, 6 min walk (CAM), SBP, BMI, LDL, HDL, uric acid, triglycerides, glucose and cholesterol total. In linear regressions, CS was associated with glucose (B:12.80; CI:9.31/20.50; *p* < 0.001; *R*^2^:0.45), light PA (B:3.10; CI:2, 41/4.76; *p* < 0.001; *R*^2^:0.57), NAF by accelerometer (B:8.21; CI:6.74/10.02; *p* < 0.001; *R*^2^:0.62), vigorous PA (B:794.03; CI:682.11/908.2; *p* < 0.001; *R*^2^:0.70), LDL (B:13.28; CI:7.45/16.75; *p* < 0.002; *R*^2^:0.71) and 6 min walk (B:3.39; CI:2.96/8.75; *p* < 0.004; *R*^2^:0.73). NAF was associated with mild PA (B:0.246; CI:0.130/0.275; *p* < 0.001; *R*^2^:0.624), moderate PA (B:0.763; CI:0.567/0.924; *p* < 0.001; *R*^2^:0.745), glucose (B:−0.437; CI:−0.789/−0.124; *p* < 0.001; *R*^2^:0.782), CAM (B:2.223; CI:1.872/4.985; *p* < 0.002; *R*^2^:0.989) and CS (B:0.253; CI: 0.189/0.512; *p* < 0.001; *R*^2^:1.94). The NAF can enhance CS. Build a new look at how these variables are independent but dependent simultaneously, being able to influence the quality of health when this dependence is denied.

## Highlights

- Level of physical activity and sedentary behavior are independent predictors of mortality.
- Sedentary behavior and physical activity level were calculated by accelerometry.
- The intensity of physical activity can enhance sedentary behavior.
- The level of physical activity can enhance sedentary behavior.
- Sedentary behavior and a physically active state coexist and are independent of health factors.
- Recommendations about increasing the level of physical activity should be accompanied by instructions to decrease sedentary behavior.

## 1. Introduction

The health benefits associated with sufficient and regular practice of moderate to vigorous physical activity (PA), as well as the risks arising from physical inactivity or insufficient levels of practice, are widely established and documented in the literature, contributing to the minimum recommendations for PA for health and for a significant paradigm shift in the area of PA and health, which occurred in the 1990s (1). The paradigm shift went from being a physically active person—to achieve health benefits, changes in physical fitness levels would be necessary—to an additive model, valuing the process of being physically active—health benefits can be achieved from a physically active lifestyle, even if there are no changes in people’s physical fitness (1, 2).

Since 1990, minimum PA recommendations for health have been suggested. Recently, the World Health Organization recommended a minimum interval of 150 to 300 min per week of PA at moderate to vigorous intensity or 75 to 150 min of intense activity per week. In parallel, evidence emerging from prospective epidemiological studies has shown that prolonged sedentary behavior (SB) promotes deleterious health effects, which may be independent of the practice of PA (2, 3). In this sense, it began to reflect that SB may be an essential health risk factor and that increasing the practice of moderate to vigorous intensity PA may not be enough to achieve the full benefits for health and quality of life (2).

PA has been characterized as any bodily movement produced by skeletal muscles which result in energy expenditure above resting levels (4). This behavior includes all daily activities, whether at work, leisure, or other activities such as: walking, eating, dressing, going upstairs, and cleaning the house. SB, on the other hand, characterizes a set of activities performed in a sitting position, which presents an energy expenditure close to resting/baseline values (1.0–1.5 MET) (watching television, using the computer, playing video games, being idle chatting with friends, talking on the phone, among other similar activities) (5, 6).

In this context, there is the possibility of a person having a sedentary behavior and being physically inactive or a physically active person (for example, walking for 60 min five or more days a week) and having a high SB time. That is, SB and PA can coexist and are shown to be independent health factors (1).

Systematic reviews of prospective epidemiological studies have demonstrated a positive relationship between prolonged SB time and all-cause death and death from cardiovascular and metabolic diseases (2, 3), regardless of PA practice. These studies demonstrated that SB is associated with biomarkers of cardiovascular and metabolic diseases, as well as risk factors for these diseases (waist circumference, body mass index, blood pressure, glucose), regardless of the level of PA (3).

Even in people who practice moderate to vigorous PA, prolonged SB time may promote harmful health effects, suggesting that this behavior represents a potential risk factor for people’s health (1).

SB as an independent predictor of people’s health is an emerging theme. The evidence available has important implications for public health and research in this area, as it suggests that SB is becoming a crucial independent health risk factor (1, 3, 4).

However, despite the literature showing it as an independent factor and presenting a range of studies on sedentary behavior and physical activity level, these studies do not address their interaction. This study wants to start understanding the extent to which sedentary behavior can be seen as a recuperative attitude after training, and from what point it starts to harm health. Build a new look at how these variables are independent in health, but dependent on each other at the same time, being able to influence the quality of health when this dependence is denied.

Thus, this study aimed to investigate the bidirectional relationship between PA level and SB, as well as their effects on a range of anthropometric, blood pressure, physical, and biochemical variables, in a sample of older women between the ages of 60 and 70 years, before and after a 14 week physical training program.

## 2. Methods

This randomized clinical trial was described following the Consolidated Standards of Reporting Trials (CONSORT) guidelines (7). The experimental procedures were submitted and approved by the Ethics Committee for Research with Human Beings of the School of Physical Education and Sport of Ribeirão Preto, University of São Paulo (CAAE: 63681517.3.0000.5659, March 24th 2017). Participants who agreed to participate in the study signed a consent form attesting to their participation in the research. The study is registered in the Brazilian Clinical Trials Registry (ReBEC) (protocol number: RBR-8hqwmx).

### 2.1. Study design

**Figure 1:**
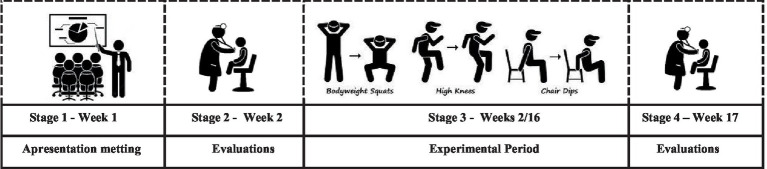
Study design: illustration from research stages.

The recruitment of participants was done through outreach in local media and social networks. Before the first evaluation, the participants were invited to a presentation meeting, where they received information about the purpose of the research and details about the test protocol—they also signed the informed consent form at the end of the session. After this stage, the participants were randomized, by a blinded researcher, into three groups: multicomponent training (MT), multicomponent training plus flexibility training (MTF), and control group (CG). This sample size was calculated using the Gpower 3.1.9.7 software with a 149 statistical power of 80%, a sampling error of 5%, and a confidence level of 95%, about the 150 estimated population of older women in Ribeirão Preto. The training lasted 14 weeks (Figure 1).

### 2.2. Participants

To be included in the study, participants had to be postmenopausal women aged 60 to 70 years, have a medical certificate of release to practice physical activity, and be physically inactive according to a score < 9.11 on the modified Baecke questionnaire for the older adults (MBQE) (8). The exclusion criteria were: having diseases and/or functional limitations (motor, auditory and visual deficits) that would prevent the tests and proposed physical training from being carried out and missing more than 25% of the physical training sessions.

### 2.3. Interventions

#### 2.3.1. Multicomponent training

The training was applied to develop coordinative motor skills and conditioning motor skills twice a week on non-consecutive days, lasting 90 min per session, divided into 15 initial minutes of warm-up, balance, coordination motor skills and games, 35 min of muscle strength training, 35 min of aerobic activities, and a final 5 min of relaxation (9, 10). Training intensity was monitored using the Borg scale adapted by Foster et al. (11, 12), to progressively track perceived exertion in values from 3 to 10, on a scale from 0 to 10 (1st and 2nd weeks: 3 to 4; 3rd to 5th week: 4 to 6; 6th to 8th week: 6 to 7; 9th to 11th week: 7 to 8; 11th to 14th week: 8 to 10), representing moderate to high-intensity physical exercise. This training emphasized strengthening the following muscles: rectus abdominis, external oblique abdominis, internal oblique abdominis, transversus abdominis, gluteus, abductors, knee flexors and extensors, deep neck flexors, serratus, rhomboids, trapezius, internal rotator cuff, and external and paraspinal or erector spinae.

#### 2.3.2. Individualized flexibility training

The flexibility capacity was trained through the active stretching method with accessories, following the protocol proposed by Kokkonen et al. (13), which follows the recommendations of the U.S. Department of Health and Human Services (14) concerning volume and intensity. Participants were separated into groups for stretching aimed at postural changes (hip flexor muscles, spine extensors, scapular elevators, and protractors), emphasizing individual needs identified after postural analysis. The training protocol contained three levels of exercise complexity for each postural compensation, with a new complexity being added every 4 weeks. The intensity and volume protocol was divided into four levels with the progression of stretching time and pain perception by the pain scale (15). The training was performed twice a week following the training progression as suggested in the Table 1 in the article (quote my article on flexibility).

**Table 1:** Protocol of flexibility training proposed in the intervention.

	Level 1	Level 2	Level 3	Level 4
Week of intervention	1–2	3–6	7–10	11–14
Duration of session	20′	30′	40′	50′
Time under tension	10″	15″	20″	25″
Interval between series	10″	15″	20″	25″
Series per exercise	2	3	4	5
Pain level^a^	1 a 3	2 a 4	4 a 6	6 a 8
Exercises per body region^b^	2	3	3	4
Weekly dose^c^	2,400″	3,600″	4,800″	6,000″

a Numeric visual/verbal scale of pain from 0 to 10.

b 8 body regions were worked in each individual—initial evaluations of each participant were considered for choice of these regions.

c Weekly dose (seconds) = duration of the session (min) * 2 (sessions/week) * 60 (seconds/min).

### 2.4. Assessments

The sample was characterized using a questionnaire prepared by the researchers to analyze demographic and health data. Systolic (SBP) and diastolic (DBP) blood pressure were measured using a previously calibrated automatic digital blood pressure gauge (OMRON^®^, model HEM-7113, SBH, 2010), and anthropometric measurements were taken of body mass (kg), height (cm), waist circumference (cm), and body mass index (kg/m^2^). The participant’s physical activity level was subjectively measured using the MBQE in conjunction with a triaxial accelerometer (GT3X-BT from ActiGraph) - the participants were instructed to use the device for 1 week, 4 days a week, and 1 day weekend was considered for the calculation. The intensities of the activities were those stipulated by Freedson et al. (16)

### 2.5. Motor skills assessments

The senior fitness test (SFT) assessed functional capacity and obtained normative values. Based on this battery of tests, the assessment of aerobic resistance [6 min walk (6 MW)] was used according to age group since this variable is one of the main variables for improving health-related physical fitness.

### 2.6. Statistical analysis

The data obtained were organized in a double-entry database using Excel®, version 2013, and the statistical program SPSS^®^, version 20.0. The per protocol (PP) method was used for the analysis, including only those participants who followed the full protocol, including weekly physical training sessions, and those participants who had all completed assessments. The analysis did not include seniors who missed more than 25% of the training sessions.

Data are presented as mean and standard deviation. The Kolmogorov–Smirnov test was used to assess data normality, and variances were analyzed using the Levene test. The analysis of training comparisons was performed using the statistical method with repeated measures ANOVA two-way, with Tukey’s post-hoc. The effect size was calculated by Cohen’s *d*, with values from 0.5 to 0.79 representing medium effect, values between 0.8 to 1.3 significant effect, greater than 1.3 enormous effect—numbers below 0.5 were considered a small effect.

Multiple linear regression was performed using the relative delta of the studied variables. The delta variation (Δ) (Δ = post-training − pre-training/pre-training) between the pre-and post-intervention moments was used to quantify the alteration of the quantitative variables. Multiple linear regression was used to analyze the interactions between sedentary behavior, physical activity level, age, socioeconomic status, MBQE, BMI, MAC, systolic and diastolic blood pressure, LDL, HDL, uric acid, triglycerides, glucose, and cholesterol. The delta variation was used in almost all the variables studied, and the age variable did not have a delta calculation. Sensitivity analysis, which involves the use of the delta of variables, is commonly employed in multiple linear regression statistical analysis. This technique is helpful as it enables researchers to evaluate the robustness of the analysis results.

In the multiple linear regression model, the data phase-in method was used to calculate the adjusted estimate (*β*) with a 95% confidence interval (95% CI). *R*^2^ was analyzed to verify the percentage of determination of the coefficient of variation, which explains the model. Multiple linear regression analysis was performed considering the level of physical activity by accelerometer and sedentary behavior, as dependent variables and MBQE questionnaire, physical activity (PA) light, moderate, vigorous and CS by accelerometry, 6 min walk (CAM), SBP, BMI, LDL, HDL, uric acid, triglycerides, glucose and total cholesterol as predictors. Data were analyzed using the statistical program Statistical Package for the Social Sciences (SPSS)^®^ version 20.0.

## 3. Results

**Figure 2:**
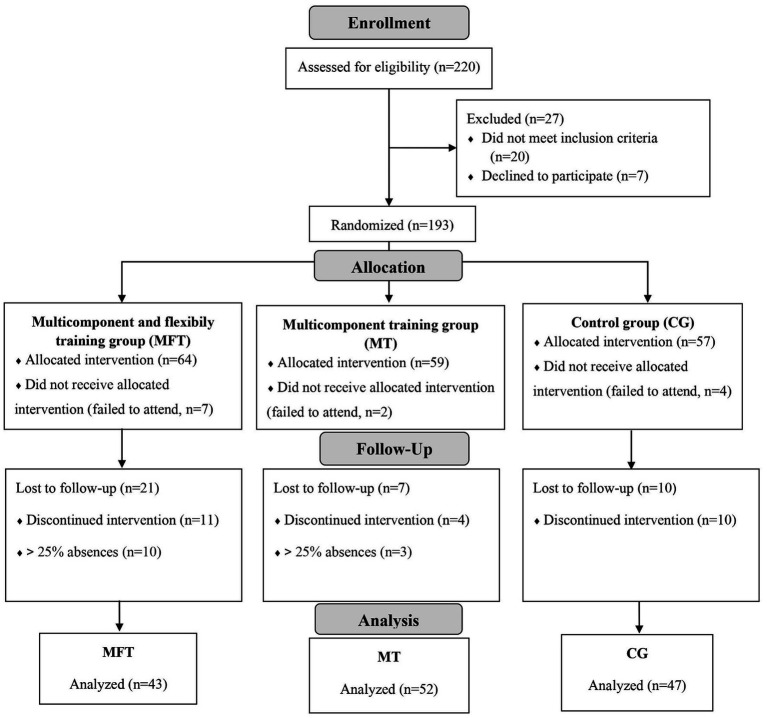
CONSORT 2010 flow diagram.

In the analyses, 43 women were included in the MFT group, 52 in the MT group, and 47 in the CG, as presented in Figure 2.

There was no statistical difference between the groups in the means for age (63.4 ± 5.6, considering all groups). A time effect is noted in the body mass variable (*F* = 8.131; *p* = 0.006), which was lower than the baseline after the intervention, and in the physical activity level evaluated by the questionnaire (*F* = 4.201; *p* = 0.010), with an increase between the pre- to the post-intervention moments.

There was a group and time interaction in the physical activity level evaluated using the accelerometry variable (*F* = 3.781; *p* = 0.016), observing an improvement from the pre- to the post-moments in both variables only in the MFT group—the MT group presented an improvement in the accelerometry between both evaluations. There was also a group and time interaction for the SBP (*F* = 3.095; *p* = 0.035) and DBP (*F* = 13.729; *p* < 0.001) variables, with a reduction in the SBP observed only in the MFT group—the DBP varied only in the CG, presenting an increase from the pre- to the post-experimental period moments. We also watched this interaction for Sedentary behavior (*F* = 10.027; *p* = 0.003), with increased results in all groups, showing a more extraordinary accentuation in the MTF group. Finally, a time and group interaction were also observed in the level of physical activity variable, being light Level (*F* = 51.59; *p* < 0.001), moderate Level (*F* = 12.08; *p* = 0.001), and vigorous Level (*F* = 17.67; *p* = 0.048), with an increase in all groups, with emphasis on the MTF concerning the other groups between the pre-and post-intervention moments (Table 2).

**Table 2:** Characterization of the participants at the pre and post experimental period moments.

	MT (*n* = 52)			MFT (*n* = 43)			CG (*n* = 47)		
	Pre	Post	Effect size	Pre	Post	Effect size	Pre	Post	Effect size
Age (years)	63.1 ± 5.5			63.4 ± 5.6			64.0 ± 4.9		
Body mass (kg)^b^	75.1 ± 14.4	73.8 ± 12.8	−0.096	74.7 ± 16.0	72.1 ± 15.8	−0.164	76.1 ± 18.8	79.3 ± 10.0	0.222
BMI (kg/m^2^)^a^	29.4 ± 5.0	29.2 ± 4.7	−0.041	28.4 ± 4.7	27.9 ± 5.3	−0.100	25.3 ± 2.7	28.4 ± 4.1^$#*^	0.912
Height (m)	1.56 ± 7.0			1.59 ± 6.9			1.58 ± 7.80		
SBP (mmHg)^a^	131.9 ± 21.3	129.0 ± 10.2	−0.184	129.2 ± 18.8	123.5 ± 15.4^*^	−0.333	135.6 ± 17.9	136.5 ± 12.4^#^	0.059
DBP (mmHg)^a^	76.8 ± 10.6	76.8 ± 8.3	0.000	76.6 ± 10.6	71.9 ± 10.0	−0.456	73.8 ± 10.1	79.1 ± 6.3^#*^	0.646
MBQE (scores)^b^	6.9 ± 5.3	11.7 ± 7.5	0.750	6.3 ± 5.4	12.6 ± 5.0	1.212	6.4 ± 5.0	8.6 ± 7.6	0.349
PA (accelerometer, counts)^a^	382 ± 1,361	877 ± 401^*^	0.562	386 ± 176	961 ± 401^*$^	1.993	348 ± 171	592 ± 365^#^	0.911
Light level of physical activity (accelerometer, counts)	2,358 ± 661	3,840 ± 2524^*^	0.803	2,410 ± 754	4,863 ± 2149^*$^	1.523	2,287 ± 545	2,667 ± 2,282^$#*^	0.232
Moderate level of physical activity (accelerometer, counts)	65 ± 77	231 ± 208^*^	1.058	66 ± 87	242 ± 228^*$^	1.101	63 ± 53	214 ± 199^$#*^	1.036
Vigorous physical activity level (accelerometer, counts)	0.16 ± 0.57	5.14 ± 3.03^*^	2.284	0.19 ± 0.66	7.48 ± 2.75^*$^	3.645	0.21 ± 0.67	0.52 ± 0.72^$#*^	0.445
Sedentary behavior (accelerometer, counts)	5,851 ± 1,453	16,415 ± 25819^*^	0.577	5,975 ± 1,310	21,975 ± 31758^*$^	0.711	5,558 ± 1739	7,588 ± 3,609^$#*^	0.716

MT, multicomponent training; MFT, multicomponent and flexibility training; CG, control group; BMI, body mass index; SBP, systolic blood pressure; DBP, diastolic blood pressure; WC, waist circumference; HC, hip circumference; BF, body fat; MBQE, modified Baecke questionnaire for the older adults; PA, physical activity.

a Interaction between time and group (*p* < 0.05).

b Time effect (*p* < 0.05).

It was possible to notice that the MT group had an effect size above moderate (>0.50) in three variables, namely the PAL-MBQE, accelerometry, and Sedentary behavior by accelerometry, presented one variable with a strong effect size (>0.8), light level of physical activity, and two variables with an extreme effect size (>1.3)—moderate and vigorous level of physical activity. The MFT group, on the other hand, presented one variable with a strong effect size (>0.8), a sedentary behavior variable, and five variables with an extreme effect size (>1.3)—in the variables of the level of physical activity by accelerometry and questionnaire, and the levels light, moderate and vigorous of physical activity. The CG group had two variables with a moderate effect size (>0.50), diastolic blood pressure and sedentary behavior, two variables with a strong effect size (>0.8), BMI, and physical activity level by accelerometry, and one variable with an extreme effect size (>1.3), Moderate level of physical activity.

The comparison of the mean and standard deviation of the biochemical variables in the pre- and post-intervention moments in the groups was observed. There was no statistical difference between groups in mean uric acid levels. There was interaction between group and time in the variables of glucose, total cholesterol, triglycerides, HDL cholesterol, and LDL cholesterol, showing improvements in the MFT group concerning the other groups from the pre- to post-intervention moment and worsening in the CG group. All biochemical variables had an effect size below moderate (<0.050) (Table 3).

**Table 3:** Biochemical analysis of the participants at the pre and post experimental period moments.

	MT (*n* = 52)			MFT (*n* = 43)			CG (*n* = 47)		
	Pre	Post	Effect Size	Pre	Post	Effect Size	Pre	Post	Effect Size
Uric acid (mg/dL)	3.9 ± 1.5	3.6 ± 1.2	0.220	4.4 ± 1.5	3.7 ± 1.3	0.498	4.3 ± 1.6	4.8 ± 2.0	0.276
Glucose (mg/dL)	105.4 ± 28.7	100.6 ± 20.1^b^	0.193	106.7 ± 26.6	95.9 ± 17.0^b^^,^^d^	0.483	100.9 ± 23.1	103.7 ± 26.2^b^^,^^c^^,^^d^	0.113
Total Cholesterol (mg/dL)	179.3 ± 37.1	175.4 ± 39.2^b^	0.102	211.4 ± 53.4	205.4 ± 44.7^b^^,^^d^	0.121	195.7 ± 50.3	201.6 ± 47.6^b^^,^^c^^,^^d^	0.120
Triglycerides (mg/dL)	117.7 ± 57.5	118.4 ± 48.2^b^	0.0131	142.6 ± 60.4	130.6 ± 69.6^b^^,^^d^	0.184	122.4 ± 57.4	137.4 ± 81.7^b^^,^^c^	0.212
HDL Cholesterol (mg/dL)	50.3 ± 13.8	49.2 ± 15.0^b^	0.076	57.2 ± 15.7	52.7 ± 17.5^b^^,^^d^	0.270	49.8 ± 16.5	53.5 ± 16.2^b^^,^^c^	0.226
LDL Cholesterol (mg/dL)	105.5 ± 33.5	102.5 ± 37.2^b^	0.084	125.6 ± 48.4	122.7 ± 42.9^b^^,^^d^	0.063	119.5 ± 48.4	122.1 ± 44.3^b^^,^^c^^,^^d^	0.056

MT, multicomponent training; MFT, multicomponent and flexibility training; CG, control group. Bold: effect size ≥ 0.50.

a Time effect (*p* < 0.05).

b *p* < 0.05 in relation to the pre-intervention moment in the same group.

c *p* < 0.05 in relation to the MFT at the same moment.

d *p* < 0.05 in relation to the MT at the same moment.

Multiple linear regression was used to generate the final model, which revealed an association between sedentary behavior deltas by accelerometry and deltas of the variables glucose, light physical activity by accelerometry, total physical activity by accelerometry, vigorous physical activity by accelerometry, LDL and walking of 6 min. The general model was responsible for 45% (*R*^2^ = 0.45) in the glucose variable, light physical activity by accelerometry 57% (*R*^2^ = 0.57), total physical activity by accelerometry 65% (*R*^2^ = 0.62), vigorous physical activity by accelerometry 70% (*R*^2^ = 0.70), LDL 71% (*R*^2^ = 0.71) and 6-min walk 73% (*R*^2^ = 0.73) of the variation in the measurement of sedentary behavior (Table 4).

**Table 4:** Final model of the multiple linear regression analysis considering delta of sedentary behavior by accelerometry as dependent variable age and deltas of socioeconomic status, activity level by MBQE questionnaire, BMI, CAM, physical activity level by accelerometry, light, moderate activity and vigorous by accelerometry, systolic and diastolic blood pressure, LDL, HDL, uric acid, triglycerides, glucose, cholesterol as predictors.

Variables	Estimates	CI 95%	*p*-value	*R* ^2^
Glucose	12.80	9.31/20.50	<0.001	0.45
Light physical activity by accelerometry	3.10	2.41/4.76	<0.001	0.57
Total physical activity by accelerometer	8.21	6.74/10.02	<0.001	0.62
Vigorous physical activity by accelerometry	794.03	682.11/908.2	<0.001	0.70
LDL	13.28	7.45/16.75	0.002	0.71
CAM	3.39	2.96/8.75	0.004	0.73

Estimate (*β*) = beta, CI 95% = confidence interval, *R*^2^ proportion of variation of the dependent variable explained by the independent variables.

Another final model, generated by multiple linear regression, revealed an association between the values of the level of physical activity by the accelerometer and the variables glucose, light physical activity by the accelerometer, moderate physical activity by the accelerometer, sedentary behavior by the accelerometer, and 6 min walk. The general model was responsible for 62% (*R*^2^ = 0.624) in the light physical activity variable by the accelerometer, moderate physical activity by accelerometer 74% (*R*^2^ = 0.745), glucose 78% (*R*^2^ = 0.782), 6 min walk 98% (*R*^2^ = 0.989) and sedentary behavior by accelerometer 194% (*R*^2^ = 1.94) of the variation in the measurement of physical activity level by accelerometer (Table 5).

**Table 5:** Final model of the multiple linear regression analysis considering delta of physical activity level by accelerometry as dependent variable age and deltas of socioeconomic status, activity level by MBQE questionnaire, BMI, MAC sedentary activity by accelerometry, light, moderate and vigorous activity by accelerometry, systolic and diastolic blood pressure, LDL, HDL, uric acid, triglycerides, glucose, cholesterol as predictors.

Variables	Estimates	CI 95%	*p*-value	*R* ^2^
Glucose	−0.437	−0.789/−0.124	0.001	0.782
Light physical activity by accelerometry	0.246	0.130/0.275	<0.001	0.624
Moderate physical activity by accelerometry	0.763	0.567/0.924	<0.001	0.745
Sedentary behavior by accelerometry	0.253	0.189/0.512	<0.001	0.947
CAM	2.223	1.872/4.985	0.002	0.989

Estimate (*β*) = beta, CI 95% = confidence interval, *R*^2^ proportion of variation of the dependent variable explained by the independent variables.

## 4. Discussion

The present study sought to present the influence of PA level on SB, and conversely, on a set of anthropometric, blood pressure, physical and biochemical variables pre and post-14 week physical training in older women aged 60 to 70. It was verified a strong relationship between SB and increased glucose, increased LDL and CAM, and a strong association with vigorous physical activity, indicating that SB increases significantly with vigorous physical activity. At the same time, a slight increase is associated with light and moderate physical activity. It was observed that the increase in the level of light to moderate physical activity is related to a decrease in glucose levels and a low relation with the increase in SB.

Health variables are known to be influenced by levels of physical activity and sedentary behavior, which makes them suitable for a linear regression analysis to improve the reliability of the data. Previous studies have shown that increased sedentary behavior is associated with increased blood glucose levels (17, 18). In contrast, an increase in physical activity levels is related to a decrease in blood glucose levels (19, 20). This study also observed similar trends in health variables, providing further support for the validity of the data presented in this paper. This study contributes to this body of literature by examining the relationship between physical activity and sedentary behavior with several health variables in older women aged 60 to 70.

In addition to the above remarks, the intervention groups showed a reduction in body mass, as observed in the study of, De Paula et al. (21), in which 12 weeks of training proved sufficient to change the anthropometric profile. In this study, the intervention took place over 14 weeks, which may explain the change in this profile, but when compared to the SB, it was noticed that there was an increase in the body mass of the SB. The same behavior can be seen in the blood pressure variable, showing that both the multicomponent and multicomponent training with flexibility is practical, even if this improvement is more significant in the MFT.

There was an improvement in the levels of physical activity by accelerometer and an increase in the score of the MBQE questionnaire of the MT and MTF groups, which was not observed in the CG, as it remained without physical activity throughout the training period, as pointed out in a study previous (10).

The differential of our study is to show that sedentary behavior (SB) increases with vigorous physical activity in older women aged 60 to 70. That is, vigorous physical activity has been shown to influence this variable. Therefore, caution should be exercised when achieving fitness levels through physical exercise and taking a fresh look at SB to understand to what extent it fits into a beneficial recovery/rest period or a period of sedentary attitudes harmful to health. This point is justified because in our data, when comparing the SB in vigorous and moderate activity, the group of women who did the physical activity with moderate intensity showed a minor increase in SB.

SB refers to energy expenditure <1.5 metabolic equivalents (METs) when awake and in a sitting posture, such as time spent driving, watching screens, and studying, among other activities, while light PA represents any activity between 1.05 to 2.99 METs and moderate to vigorous physical activity (MVPA) ≥3.0 METs (22).

The systematic review carried out by Saunders et al. (23) presents data that indicate that high levels of SB increase the risk of mortality from various causes and points out that there is strong evidence of a dose-response relationship, which would present a significant relationship with health. Even though mortality is essential for public health, it does not represent the totality of human health and well-being, as other indicators are just as significant.

Ekelund et al. (24) state that a shorter MVPA time with a longer SB time is associated with a higher risk of all-cause mortality and evidence measured by accelerometry, which indicates that the increase in MVPA time for a variation between 30 to 40 min per day presents a positive association when compared to SB and mortality.

This phenomenon, reported in this study, is known as the “amplification of sedentary behavior” and is thought to be due to the need for physiological recovery after exercise. Physical activity is essential to maintain health and reduce the risk of chronic diseases. Physical training is a common intervention to promote increased physical activity, especially in older people. The results of this study corroborate recent studies that have demonstrated this trend of increasing sedentary behavior and physical activity level.

A study conducted with older adults revealed that those who practiced high levels of physical activity had higher levels of fatigue and lower energy levels than those who practiced moderate physical activity (25). The authors further concluded that this might lead individuals to adopt a more sedentary behavior to conserve energy and recover from exercise. In the results of this study, we noticed that older people who practiced moderate to vigorous activity increased sedentary behavior more.

At moderate to vigorous physical activity levels, the amplification of sedentary behavior may be due to the need for physiological recovery after exercise. Exercise stresses the body, leading to inflammation, muscle damage, and other physiological changes. These changes require time to repair and recover, which can result in increased sedentary behavior.

For example, a study of older adults found that those who engaged in high levels of physical activity had higher levels of C-reactive protein (CRP), a marker of inflammation, than those who engaged in moderate physical activity (26). This suggests that the high levels of physical activity caused increased inflammation, leading to increased sedentary behavior as the body recovers. In the data from this study, we saw that the vigorous level of activity had a broader increase in sedentary behavior, which aligns with the data (26) presented.

The amplification of sedentary behavior has important implications for public health. Sedentary behavior is associated with numerous health risks, including obesity, cardiovascular disease, and type 2 diabetes (27). In this study, there was a strong relationship between BS and increased blood glucose, and increased LDL. These factors can contribute to the development this diseases. Therefore, it is essential to understand the factors contributing to the increase in sedentary behavior concerning physical training interventions and thus improve training approaches to maximize results.

These outcomes corroborate the increase in SB observed in our study when there was an increase in moderate to vigorous physical activity and a reduction in mild PA. Even though SB is associated with low PAL, there are indications that the increase in moderate to vigorous physical activity in the older adults generated a compensatory behavior and, consequently, the need for increased recovery time in older women after the training periods. Vigorous physical activities are beneficial in the age group studied since there was an improvement in biochemical factors, but they increase SB Thus, when there is an increase in PAL, one must pay attention to the moment when the increase in SC means a possible amplification of the need for recovery from the physical activity performed or a harmful adoption of SC.

There is a need for further studies investigating this topic. Paying more attention to the behavior of the older adults after a period of physical exercise may be a possible strategy for improving results.

## 5. Conclusion

In this study, it was concluded that there is a possible relationship between an increase in the level of physical activity and an increase in sedentary behavior in older adults. Thus, it becomes important to take a closer look at the sedentary behavior variable after starting the practice.

## Data availability statement

The raw data supporting the conclusions of this article will be made available by the authors, without undue reservation.

## Ethics statement

The studies involving human participants were reviewed and approved by Ethics Committee for Research with Human Beings of the School of Physical Education and Sport of Ribeirão Preto, University of São Paulo (CAAE: 63681517.3.0000.5659, March 24th 2017). The patients/participants provided their written informed consent to participate in this study.

## Author contributions

GO and AS: formal analysis, conceptualization, methodology, visualization, investigation, and writing—original draft preparation. CB: writing—review and editing, original draft preparation, formal analysis, and conceptualization. GR, KR, CF, AC, and TF: conceptualization, methodology, and visualization. GG and CJ: formal analysis, visualization, supervision, conceptualization, and writing—review and editing. All authors contributed to the article and approved the submitted version.

## Funding

This work was supported by the Fundação de Amparo à Pesquisa do Estado de São Paulo FAPESP (FAPESP—case no. 2017/21361-2), the Conselho Nacional de Desenvolvimento Científico e Tecnológico (CNPq—case nos. 485045/2013-3 and 141720/2017-4), and the Coordenação de Aperfeiçoamento de Pessoal de Nível Superior—Brazil (CAPES—finance code 001).

## Conflict of interest

The authors declare that the research was conducted in the absence of any commercial or financial relationships that could be construed as a potential conflict of interest.

## Publisher’s note

All claims expressed in this article are solely those of the authors and do not necessarily represent those of their affiliated organizations, or those of the publisher, the editors and the reviewers. Any product that may be evaluated in this article, or claim that may be made by its manufacturer, is not guaranteed or endorsed by the publisher.
